# Inhibition of mirtazapine metabolism by Ecstasy (MDMA) in isolated perfused rat liver model

**DOI:** 10.1186/s40199-017-0183-z

**Published:** 2017-06-28

**Authors:** Sanaz Jamshidfar, Yalda H. Ardakani, Hoda Lavasani, Mohammadreza Rouini

**Affiliations:** Biopharmaceutics and Pharmacokinetic Division, Department of Pharmaceutics, Faculty of Pharmacy, Tehran University of Medical Sciences, Tehran, Iran

**Keywords:** Mirtazapine, Ecstasy, Metabolism, Isolated perfused rat liver model

## Abstract

**Background:**

Nowadays MDMA (3,4-methylendioxymethamphetamine), known as ecstasy, is widely abused among the youth because of euphoria induction in acute exposure. However, abusers are predisposed to depression in chronic consumption of this illicit compound.

Mirtazapine (MRZ), an antidepressant agent, may be prescribed in MDMA-induced depression. MRZ is extensively metabolized in liver by CYP450 isoenzymes. 8-hydroxymirtazapine (8-OH) is mainly produced by CYP2D6. N-desmethylmirtazapine (NDES) is generated by CYP3A4.

MDMA is also metabolized by the mentioned isoenzymes and demonstrates mechanism-based inhibition (MBI) in association with CYP2D6. Several studies revealed that MDMA showed inhibitory effects on CYP3A4.

In the present study, our aim was to evaluate the impact of MDMA on the metabolism of MRZ in liver.

Therefore, isolated perfused rat liver model was applied as our model of choice in this assessment.

**Methods:**

The subjects of the study were categorized into two experimental groups. Rats in the control group received MRZ-containing Krebs-Henselit buffer (1 μg/ml). Rats in the treatment group received aqueous solution of 1 mg/ml MDMA (3 mg/kg) intraperitoneally 1 hour before receiving MRZ. Perfusate samples were analyzed by HPLC.

**Results:**

Analyses of perfusate samples showed 80% increase in the parent drug concentrations and 50% decrease in the concentrations of both metabolites in our treatment group compared to the control group.

In the treatment group compared to the control group, AUC_(0–120)_ of the parent drug demonstrated 50% increase and AUC_(0–120)_ of 8-OH and NDES showed 70% and 60% decrease, respectively.

Observed decrease in metabolic ratios were 83% and 79% for 8-OH and NDES in treatment group compared to control group, respectively.

Hepatic clearance (CL_h_) and intrinsic clearance (Cl_int_) showed 20% and 60% decrease in treatment group compared to control group.

**Conclusion:**

All findings prove the inhibitory effects of ecstasy on both CYP2D6 and CYP3A4 hepatic isoenzymes.

In conclusion, this study is the first investigation of MRZ metabolism in presence of MDMA in isolated perfused rat liver model.

**Graphical abstract:**

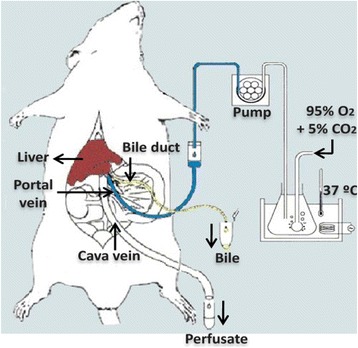

## Background

Nowadays, hallucinogens and stimulants are profoundly abused by the youth due to their primary effects to elevate energy levels, to induce euphoria, and to reach higher levels of pleasure and empathy. MDMA (3,4-methylenedioxymethamphetamine), known as ecstasy, is one of these illicit drugs that acts as both releaser and reuptake inhibitor of central neurotransmitters and causes a large number of adverse effects not only in acute encounter but also in long-term abuse [1].

Major metabolic pathways involved in the bioactivation of MDMA are N-dealkylation and O-demethylenation. N-dealkylation of MDMA, catalyzed by CYP2B6, CYP1A2, and CYP2C19, produces the metabolite 3,4-methylendioxyamphetamine (MDA). O-demethylenation of MDMA and MDA, mediated by CYP2D6, CYP3A4, and CYP2C19, produces 3,4-dihydroxymethamphetamine (HHMA) and 3,4-dihydroxyamphetamine (HHA), respectively. HHMA and HHA metabolism generates 4-hydroxy-3-methoxymethamphetamine (HMMA) and 4-hydroxy-3-methoxyamphetamine (HMA) by the activity of catechol-O-methyltransferase (COMT) [2, 3].

MDMA demonstrates non-linear pharmacokinetics; the non-linearity is due to mechanism-based inhibition (MBI), observed in chemical structures containing methylendioxy group [1, 4]. Previous studies revealed that MDMA exhibited MBI in association with CYP2D6 isoenzyme. It is hypothesized that CYP2D6 forms an orthoquinone intermediate with methylendioxyphenyl ring of MDMA. This complex attacks macromolecular structures as a nucleophile and interferes with their functions [4, 5]. These events result in irreversible inhibition of CYP2D6 isoenzyme, which resembles CYP2D6 inhibition by paroxetine [4, 6]. This phenomenon involves most of CYP2D6 isoenzymes and is demonstrated within 1 h of MDMA administration [7].

Chronic abuse of MDMA is associated with a wide variety of complications such as serotonergic and dopaminergic nerve deterioration, cognitive disorders, and psychological problems [8]. Among these adverse effects, depression is one of the most common psychiatric problems associated with long-term consumption of ecstasy. Treatment of MDMA-induced depression is, therefore, of great value.

Mirtazapine (MRZ), a piperazinoazepine compound, belongs to noradrenergic and specific serotonergic antidepressants (NaSSA) [9]. Its pharmacological activity is virtually associated with presynaptic-α2 receptor blockade, which results in an increase in both serotonin and norepinephrine levels and contributes to antidepressant activities [10]. In clinical practice, MRZ is particularly indicated for treatment of major depressive disorder (MDD) [11].

Previous clinical studies have shown that MRZ is superior to placebo and some selective serotonin reuptake inhibitors (SSRIs) such as fluoxetine. MRZ also has equal efficacy as some tricyclic antidepressants (TCAs) such as amitriptyline and clomipramine in treatment of depression. Moreover, MRZ is better tolerated during the treatment process since it does not induce nausea and has less anticholinergic side effects. MRZ exhibits more rapid onset of action compared with TCAs and SSRIs [11].

MRZ demonstrates linear pharmacokinetics in the therapeutic dosage regimen (15 to 80 mg/day). Good absorption via oral route and elimination half-life of 20 to 40 h, that is in favor of once daily dosing, are of notable pharmacokinetic parameters of MRZ [12].

MRZ is mostly excreted in urine and is extensively metabolized in liver by means of CYP450 isoenzymes. CYP3A4, CYP2D6 and CYP1A2 are involved in biotransformation of MRZ. 8-hydroxymirtazapine (8-OH), the major metabolite, is mainly produced by CYP2D6 and to a lesser extent by CYP1A2. N-desmethylmirtazapine (NDES), the only pharmacologically active metabolite, and N-oxidemirtazapine are generated by CYP3A4 [13, 14].

In this study we focused on the possible interaction between MRZ and MDMA, considering both CYP2D6 and CYP3A4 isoenzymes. We assessed the impact of MDMA on the metabolism of MRZ in liver. Therefore, isolated perfused rat liver model was applied as our model of choice in this assessment.

## Materials and methods

### Materials

MRZ, 8-OH and NDES were kindly supplied by Mario Georgi (University of Pisa, Italy).

MDMA.HCl powder was synthesized in Medicinal Chemistry Department of Tehran University of Medical Sciences according to the previously reported [15] method that showed the acceptable purity compared to standard sample purchased from Lipomed Pharmaceutical (Switzerland). The structure was confirmed by IR, H-NMR and Mass spectra.

HPLC grade acetonitril and methanol, and analytical grade salts such as potassium dihydrogen phosphate were all from Merck (Darmstadt, Germany).

### Standard solutions

**Fig. 1:**
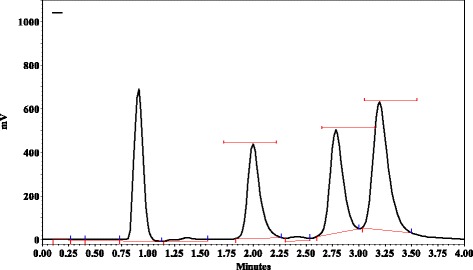
Chromatogram of standard solution (40 ng/ml of 8OHand 80 ng/ml of NDES and MRZ). Retention time:(8-OH: 2 min, NDES: 2.75 min, MRZ: 3.25 min)

Primary stocks of MRZ and its two major metabolites were prepared by dissolving pure powders in methanol to make concentration of 1 mg/ml. Final dilution for QC and calibration samples was done using Krebs-Henselit buffer. MRZ and NDES concentrations were between 5 and 150 ng/ml. Concentrations used for 8-OH were between 2.5 and 75 ng/ml (Fig. 1). All the above solutions were kept at 4 °C. Aqueous stock of MDMA (1 mg/ml) was prepared by dissolving in double distilled water.

### Animals

Twelve healthy male Sprague-Dawley rats (divided into two experimental groups called control and treatment) weighting between 250 and 300 g were applied in this assessment. They were kept under 12-h light-dark cycle with controlled environment temperature and without any limit in access to standard laboratory chow and water.

The present study was approved by the Institutional Review Board of Tehran University of Medical Sciences. Ethical approval code number was [253066].

### Rat liver perfusion

The rats were anesthetized via intraperitoneal (IP) injection of xylazine/ketamine mixture (15/75 mg/kg). Cannulation of portal vein and inferior vena cava was done using previously heparinized intravenous catheters (guage of 16, 18 respectively). After inserting catheters, the Krebs-Henselit buffer (118 mM NaCl, 4.5 mM KCl,2.75 mM CaCl_2_, 1.19 mM KH_2_PO_4_, 1.18 mM MgSO_4_, and 25 mM NaHCO_3_) adjusted to the physiological pH (using 95% O_2_/5%CO_2_) was passed through the portal vein employing peristaltic pump set on the constant flow rate of 500 ml/h for 10 min. Then MRZ-containing medium (inlet concentration of 1 μg/ml) was delivered into the portal vein for 120 min and the perfusate samples were collected immediately after wash and then every 10 min from the inferior vena cava to calculate outlet concentrations of parent drug and both metabolites.

Earlier rat liver perfusion studies with MRZ in our lab revealed that 8-OH metabolite of MRZ was not detectable in low parent drug concentrations while NDES was measurable even in low concentrations of MRZ, so, a 1 μg/ml concentration of MRZ was selected as exposure concentration for control (*n* = 6) and treatment (*n* = 6) group.

Previous studies showed that single dose administration of MDMA (3 mg/kg) to rats resembles the plasma concentration following the ordinary MDMA dosage taken in humans [16].

So, treatment group were received freshly prepared aqueous solution of 1 mg/ml MDMA (3 mg/kg) intraperitoneally 1 hour before receiving MRZ-containing medium (1 μg/ml) through the single pass mode of liver perfusion.

**Fig. 2:**
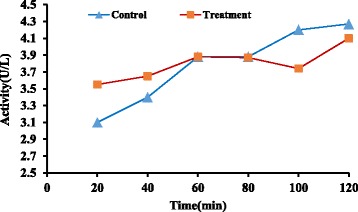
Mean profile of AST during the perfusion study in control and treatment groups (*n* = 6)

**Fig. 3:**
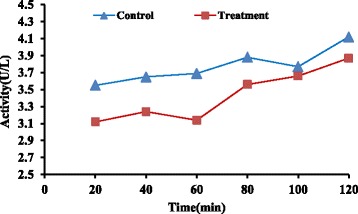
Mean profile of ALT during the perfusion study in control and treatment groups (*n* = 6)

Perfusion pressure (15 mmHg), temperature, and pH (7.4) were monitored continuously during the procedure and remained constant till the end. Liver viability tests were carried out intermittently and were passed. Normal range for AST and ALT are 0–46(U/L) and 0–49(U/L) respectively (Figs. 2 and 3).

Collected samples (in 10 min intervals) were centrifuged for 15 min at 12000 rpm and the clear solutions were separated and were stored at −20 °C until use.

### Apparatus and chromatographic condition

The chromatographic apparatus consisted of a low pressure gradient HPLC pump coupled with fluorescence detector (290 nm–370 nm), a 100μlit loop, and a Rheodyne model 7725i injector, all from Knauer (Berlin, Germany).

A Chromolith™Performance RP-8e 100 mm × 4.6 column (Knauer, Berlin, Germany) attached to a protective Chromolith™ guard cartridge RP-18e 5 mm × 4.6 mm was applied for chromatographic separation (Merck, Darmstadt, Germany).

A mixture of 0.025 M KH_2_PO_4_ buffer, adjusted to pH = 3 using ortho-phosphoric acid, and acetonitril (83:17, *v*/v) was employed as mobile phase and was delivered through the column with the constant flow rate of 2 ml/min [17]. Data acquisition and analyses were achieved by ChromGate chromatography software (Knauer, Berlin, Germany).

### Pharmacokinetic parameters

The analytes concentrations were determined using standard calibration curves. Based on concentrations of MRZ and its two metabolites, the areas under the concentration versus time curves AUC _(0–120)_ were acquired using trapezoidal rule. The metabolic ratios at different times for both metabolites were calculated using metabolite concentration divided by MRZ concentration at specific times.

Availability (F), extraction ratio (E), clearance (CL_h_) and intrinsic clearance (CL_int_) were of hepatic pharmacokinetic parameters determined in this study using following equations [18].

1 $$ \mathrm{F}=\frac{\mathrm{mean}\ \mathrm{outlet}\ \mathrm{concentration}\ \mathrm{of}\ \mathrm{MRZ}\ \mathrm{at}\ \mathrm{four}\ \mathrm{latest}\ \mathrm{samples}}{\mathrm{inlet}\ \mathrm{concentration}\ \mathrm{of}\ \mathrm{MRZ}} $$

2 $$ \mathrm{E}=1-\mathrm{F} $$

3 $$ {\mathrm{CL}}_{\mathrm{h}}=\mathrm{Q}\times \mathrm{E} $$

4 $$ {\mathrm{CL}}_{\mathrm{int}}=\mathrm{E}\times \frac{\mathrm{Q}}{\mathrm{F}} $$

Q, in the above equation, is the constant perfusion flow rate of 500 ml/h that equals 8.3 ml/min.

Since concentrations of MRZ and its two metabolites reach the plateau at four latest time intervals, mean concentration of these samples were used in the equation.

All data in this study were reported as Mean ± SD.

### Statistics

The t-test was applied in this study in order to determine differences between means of groups (*P* value <0.05).

## Results

**Fig. 4:**
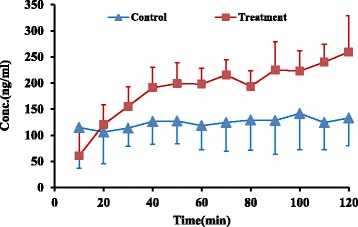
Mean MRZ concentration (±SD) vs. time in control and treatment groups (*n* = 6)

According to our results, analyses of four latest sample intervals taken from treatment group showed 80% enhancement in parent drug concentration (236.8 ± 44.7 vs 131.9 ± 56.9 ng/ml) in comparison to control group (*P* value < 0.05) (Fig. 4).

Similarly, the AUC _(0–120)_ of parent compound also showed 50% enhancement in treatment group comparing to control group (1792.5 ± 2871.7 vs 1184.8 ± 3655.2 ng.min/ml), (*P* value < 0.05) (Table 1).

**Table 1:** AUC _(0–120)_ MRZ, NDES, and 8-OH in control and treatment groups (*n* = 6)

	MRZ		8-OH		NDES	
	Control	Treatment	Control	Treatment	Control	Treatment
1	15,530.1	24,053.4	2124.9	287.5	28,525.2	593.7
2	9928.7	18,031.1	1398.9	398.6	22,505.4	12,111.8
3	16,443.6	17,675.6	1960.2	449.2	15,171.6	15,346.2
4	10,611.4	23,553.7	1674.1	415.4	18,839.8	5860.1
5	13,295.1	23,063.4	610.9	371.3	11,195.2	10,613.3
6	19,497.1	22,680.5	588.2	920.5	12,075.7	3605.2
MEAN	14,217.7	21,509.6*	1392.9	473.7*	18,052.1	8021.7*
SD	3655.2	2871.7	662.7	225.6	6650.9	5593.7
CV	25.7	13.4	47.6	47.6	36.8	69.7

*P value < 0.05, significant difference between control and treatment groups

**Fig. 5:**
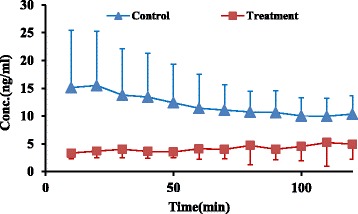
Mean 8-OH concentration (±SD) vs. time in control and treatment groups (*n* = 6)

**Fig. 6:**
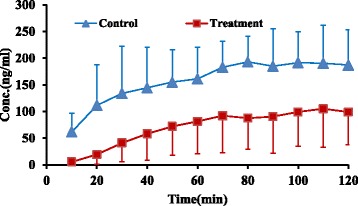
Mean NDES concentration (±SD) vs. time in control and treatment groups (*n* = 6)

**Fig. 7:**
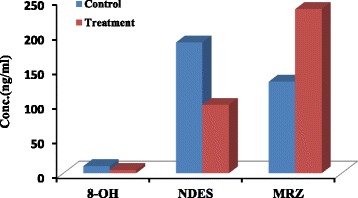
Comparative histogram of MRZ, NDES and 8-OH concentrations at four latest samples in control and treatment groups (*n* = 6)

Contrary to parent drug, concentrations of both metabolites, 8-OH and NDES, showed 50% decrease in treatment group at four latest sampling time intervals compared to control group (4.7 ± 2.9 vs 10.3 ± 3.2 ng/ml) and (98.5 ± 64.3 vs 188.6 ± 63.44 ng/ml), (*P* value < 0.05) respectively (Figs. 5, 6 and 7).

In accordance with decreasing in both metabolite concentrations in perfusate buffer at four latest sampling time intervals, the AUC _(0–120)_ of both metabolites showed 70% and 60% decrease for 8-OH and NDES respectively in treatment group in comparison to control group (473.7 ± 225.6 vs 1392.9 ± 662.7 ng.min/ml) and (8021.7 ± 5593.7 vs 18,052.1 ± 6650.8 ng.min/ml), (*P* value < 0.05) respectively (Table 1).

Based on pharmacokinetic equations, CL_h_ and CL_int_ demonstrated 20% and 60% decrease respectively in treatment group compared to control group (6.3 ± 0.4vs 7.2 ± 0.5 ml/min) and (27.7 ± 6.3 vs 63.4 ± 25.8 ml/min), (*P* value < 0.05), respectively (Table 2).

**Table 2:** Comparison of availability, extarction ratio, clearance and intrinsic clearance (±SD) in control and treatment groups (*n* = 6)

	F	E	CL_h_(ml/min)	CL_int_(ml/min)
Control	0.13 ± 0.06	0.87 ± 0.06	7.21 ± 0.47	63.4 ± 25.81
Treatment	0.24 ± 0.04	0.76 ± 0.06	6.33 ± 0.37	27.71 ± 6.26
*P* value	0.005	0.004	0.005	0.008

**Fig. 8:**
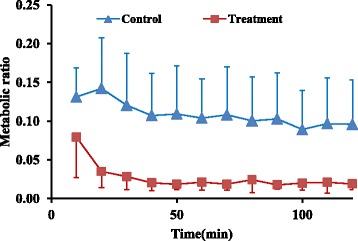
Mean metabolic ratio of 8-OH (±SD) at different time intervals in treatment and control groups (*n* = 6)

**Fig. 9:**
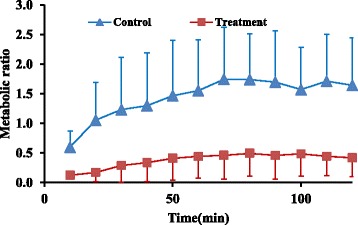
Mean metabolic ratio of NDES (±SD) at different time intervals in control and treatment groups (*n* = 6)

Metabolic ratios for 8-OH, main metabolite of CYP2D6, and NDES, main metabolite of CYP3A4, at four latest sampling time intervals showed 83% and 79% decrease respectively in treatment group compared to control group (0.02 ± 0.008 vs 0.1 ± 0.05) and (0.45 ± 0.3 vs 1.7 ± 0.8), (*P* value < 0.05), respectively (Figs. 8 and 9).

## Discussion

Hepatic metabolism appears to play an important role in the toxicity induced by MDMA consumption [19]. MDMA is metabolized by various cytochrome P450 enzymes. The primary pathway is O-demethylenation by CYP2D6; however, it has been shown that CYP3A4 is effectively involved in bioactivation of MDMA. Although several studies were focused on MDMA as a potent mechanism-based inhibitor of CYP2D6, a number of investigations proposed that inhibitory effects of MDMA on CYP3A4 could also be of great clinical value [20, 21].

Since both CYP3A4 and CYP2D6 are involved in many drugs metabolism, simultaneous use of MDMA and other substances could be of high concern.

In order to extend the desirable effects and cut down the undesirable ones such as depression or anxiety, most ecstasy users are interested in using other pharmaceutical drugs concurrently.

Previous studies have focused mostly on the pharmacodynamic interactions between MDMA, on one hand, and SSRIs and MAO inhibitors, on the other hand.

In a clinical study, after pretreatment of subjects with 20 mg/day of paroxetine (a potent inhibitor of CYP2D6 and an SSRI) for 3 days before MDMA administration, thirty hundred percent enhancement in MDMA plasma concentrations was observed. In spite of this concentration enhancement, psychological effects of MDMA were attenuated due to pharmacodynamic interactions [22].

Co-administration of moclobemide (a selective MAO A inhibitor) and MDMA caused several reported deaths due to serotonin syndrome [23].

MRZ can be prescribed in MDMA-induced depression [24]. Moreover, MDMA may be abused in patients suffering from depression who are under treatment with MRZ as a mood elevator [25]. The present study was, therefore, proposed to evaluate the pharmacokinetics of MRZ and its two main metabolites after MDMA administration.

As recent studies revealed that MBI could occur shortly after a single dose administration of MDMA, 1-hour interval was chosen between rat IP injection of MDMA and exposure of liver to MRZ.

Our findings prove the inhibitory effects of MDMA not only on CYP2D6 but also on CYP3A4 hepatic isoenzymes.

Although the inhibitory mechanism of MDMA on CYP2D6 is known as MBI, the mechanisms that have been proposed to justify the inhibitory effects of ecstasy intake on other CYPs include increased levels of neurotransmitters, impairment of mitochondrial function, oxidation of biogenic amines, and metabolic bio-activation. Among these, it has been shown that biotransformation of MDMA has a significant role in its hepatotoxicity because of its oxidative metabolites corresponding to the formation of ortho-quinones. These ortho-quinone compounds are able to enter a redox cycle that has been proposed to be the reason of cytotoxicity in several tissues such as liver, brain, kidney, and heart [2, 5–7, 20, 21, 26].

While several studies have focused on the interaction of MDMA with CYP2D6, the interaction of MDMA with CYP3A4 would also be of great clinical relevance because of the potential hepatotoxicity in MDMA abusers, which results from the reaction between oxidative metabolites and other essential intracellular macromolecules.

Another finding of this study, i.e., 80% enhancement in outlet perfusate concentration of MRZ in treatment group in comparison to control group, may propose the importance of dose adjustment in clinic. This finding is in line with two case reports [27] concerning the interactions between MRZ and fluvoxamine (an inhibitor of both CYP2D6 and CYP3A4 isoenzymes), that had reported 3 to 4 fold increase in serum concentrations of MRZ manifested by increased anxiety in patients under treatment [28].

## Conclusion

In conclusion, this study is the first investigation of the metabolism of MRZ in presence of MDMA in isolated perfused rat liver model. To our knowledge, only few studies have been carried out on this subject. Complementary studies with higher amounts of MDMA or with different time intervals between IP injection of MDMA and liver perfusion are suggested to be designed.

## Abbreviations

8-OH: 8-hydroxymirtazapine
COMT: Catechol-O-methyltransferase
HHA: 3, 4-dihydroxyamphetamine
HHMA: 3, 4-dihydroxymethamphetamine
HMA: 4-hydroxy-3-methoxyamphetamine
HMMA: 4-hydroxy-3-methoxymethamphetamine
MBI: Mechanism-based inhibition
MDA: 3, 4-methylendioxyamphetamine
MDMA: 3, 4-methylenedioxymethamphetamine
MRZ: Mirtazapine
NDES: N-desmethylmirtazapine
